# Selection of Anti-TIGIT Affibody Molecules Blocking the Interaction between TIGIT and CD155

**DOI:** 10.1021/acsomega.6c05539

**Published:** 2026-10-22

**Authors:** Linda Ehrenborg, Javad Garousi, Jie Zhang, Hans Grönlund, Torbjörn Gräslund

**Affiliations:** † Department of Protein Science, KTH Royal Institute of Technology, Roslagstullsbacken 21, 106 91 Stockholm, Sweden; ‡ Therapeutic Immune Design, Department of Clinical Neuroscience, Center for Molecular Medicine, Karolinska Institutet, Visionsgatan 18, L8, 171 77 Stockholm, Sweden

## Abstract

T cell immunoglobulin and ITIM domain (TIGIT) is an immune checkpoint receptor expressed on T and NK cells that, upon interaction with its ligand CD155, suppresses immune cell activation. Tumor cells sometimes upregulate CD155 to evade detrimental immune reactions, and TIGIT is therefore an attractive target for the development of novel checkpoint inhibitor therapies. TIGIT has also been shown to be upregulated on tumor-infiltrating lymphocytes (TILs), enabling imaging of TILs for patient stratification for immune checkpoint therapy. Here, we have selected so-called affibody molecules targeting TIGIT from a combinatorial affibody library displayed on the surface of*Escherichia coli*. The two most promising candidates had equilibrium dissociation constants (*K*_D_ values) of 2.5 and 6.5 nM, and were found to specifically interact with TIGIT-expressing cells. Furthermore, the affibody candidates were found to successfully block the interaction between TIGIT and CD155, suggesting future application as immune checkpoint inhibitors. Using an ensemble of general language models for *in silico* evolution, a variant with a 4.4 °C increased melting temperature could be derived, albeit with a 2-fold weaker affinity. The promising results justify further development of the anti-TIGIT affibodies for *in vivo* TIL-imaging and immune checkpoint therapy.

## Graphical abstract

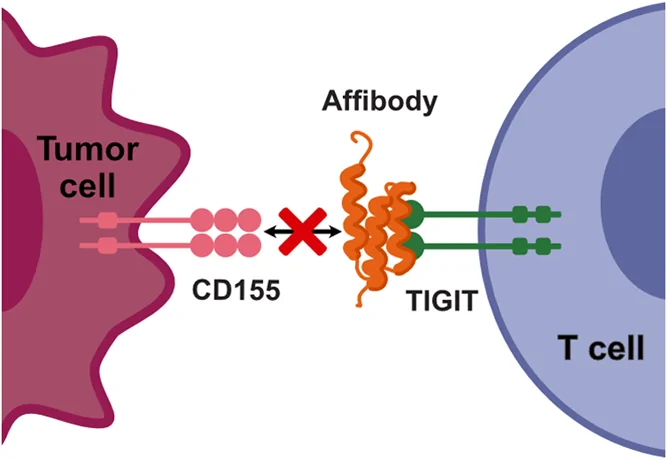

## Introduction

Immunotherapy is an area of cancer treatment that has gained traction in the past decade, particularly so-called immune-checkpoint therapy (ICT).^1^ ICT targets immune checkpoints (ICPs), which are receptors expressed on immune cells that help regulate their activity. Cancer cells can exploit ICPs by upregulating agonistic ligands, which, in turn, allow them to evade detrimental immune responses. In such cases, blocking the interaction between the ICP and its ligand on the cancer cell releases the brakes on the immune system, which reactivates an immune response to the tumor.^1,2^

The most studied ICPs are CTLA-4 and PD-1, with several antibody-based immune checkpoint inhibitors (ICIs) approved for clinical use. The CTLA-4 targeting monoclonal antibody (mAb) ipilimumab has for example been found to induce tumor regression in patients with metastatic melanoma, leading to a long-term survival of 21%,^3^ however, often accompanied by adverse side-effects.^4^ Another example is the anti-PD-1 mAb nivolumab, which was found to give patients with non-small cell lung cancer a significantly better overall response rate and progression-free survival compared to patients treated with docetaxel.^5^ However, only 10%–30% of patients respond to the treatments. Therefore, next-generation ICIs are being researched to improve overall clinical responses, with a vast number of ongoing clinical studies assessing the therapeutic effect of targeting ICPs, such as LAG-3, TIM-3, and TIGIT.^6^

One promising next-generation ICP is the T cell immunoglobulin and ITIM domain (TIGIT) receptor, also known as WUCAM, VSIG9, or Vstm3. TIGIT is expressed on the surface of CD4^+^ T cells, CD8^+^ T cells and T_regs_, as well as natural killer (NK) cells.^7–10^ The ligand with the strongest affinity for TIGIT is CD155 (*K*_D_ = 1–3 nM),^7^ which is expressed on *e*.*g*., monocytes, dendritic cells (DCs), fibroblasts, and endothelial cells. Upon activation of TIGIT by CD155 stimulation through heterotetramer formation and *cis–trans* clustering,^11^ the cytotoxicity of CD8^+^ T cells and NK cells is inhibited, which induces expression of immunosuppressive IL-10 and downregulation of the pro-inflammatory cytokine IL-12.^7,12^ TIGIT expression has been shown to exert its immunosuppressive effects both directly through T cell-intrinsic function, causing decreased T cell activation, and IL-2 production, and indirectly through modulation of cytokine production by DCs.^7,13,14^ For NK cells, it has been shown that TIGIT expression is associated with cellular exhaustion, and TIGIT blockade has been found to reverse this effect, leading to increased NK cell survival. These findings demonstrate the critical importance of NK cells in mediating the therapeutic effect stemming from TIGIT blockade.^15^ In contrast, TIGIT expression on T_regs_ enhances their immunosuppressive function,^16^ adding to the overall inhibitory effect on the immune system caused by activation of the TIGIT-CD155 axis.

Another ligand of CD155 is CD226, which instead promotes activation of the immune system.^17,18^ However, the affinity of CD155 for TIGIT is approximately 100-fold stronger compared to CD226 (*K*_D_ = 119 nM),^7^ favoring TIGIT and immunosuppression when both receptors are present. Blocking TIGIT from interacting with CD155 promotes CD226 activation, in turn leading to increased immune system activity.^19–21^

In preclinical studies, TIGIT-targeting therapeutics have shown mixed results as monotherapies, but good results in combination with other drugs.^22^ Currently, several anti-TIGIT antibodies are showing promising results in phase III clinical evaluation in combination with other treatments.^22^ For example, a greater overall antitumor response has been found for anti-TIGIT mAbs in combination with anti-PD-1/PD-L1 mAbs compared to anti-PD-1/PD-L1 monotherapies.^23^ Additionally, bispecific antibodies binding both TIGIT and PD-L1 are under development and have shown encouraging initial results.^24–26^ Taken together, TIGIT is an attractive target for the development of next-generation ICTs, with anti-TIGIT ICTs holding great promise to complement currently available immunotherapies.

Beyond therapy, quantifying TIGIT expression could be a way to assess immune system activity in tumors. For lung cancer patients, it has, for example, been found that high TIGIT expression in the tumor is correlated with a poorer prognosis and shorter median survival.^27,28^ Development of *in vivo* probes for determination of TIGIT expression may therefore become tools for diagnostic and prognostic purposes in cancer precision medicine.

Most current ICTs rely on a mAb to block the interaction between an ICP and its ligand. However, other classes of affinity proteins are also under investigation, for example nanobodies.^29^ The affibody class of alternative scaffold affinity proteins is one option for the development of new targeted therapies and *in vivo* imaging agents. They are small (58 amino acids, *M*_W_ 6.5 kDa), robust proteins arranged in a three-helix bundle structure.^30^ The small size of affibody molecules enables better tumor penetration compared to mAbs, which might, consequently, lead to an improved therapeutic effect.^31,32^ Moreover, their small size and lack of cysteines in the framework make production significantly less complex compared to mAbs, and affibody molecules are typically produced in a simple prokaryotic host cell, such as *Escherichia coli*. For some affibody molecules, it has even been demonstrated that they can be produced *in vitro* by peptide synthesis.^33^

Affibody molecules have been successfully developed against many cancer-relevant targets, including CD28, CD25, IGF1R, and EGFR.^34–37^ In these cases, variants with the ability to efficiently block ligand binding have been identified, suggesting that affibody molecules could be developed against ICPs or their ligands to serve as immune checkpoint inhibitors.

To leverage the advantages of the affibody scaffold, we have developed anti-TIGIT affibody molecules in this study, intended for future use as immune checkpoint inhibitors, as well as for the construction of probes for imaging TIGIT-expression in tumors. The two top candidates had low nanomolar affinities for TIGIT and could bind TIGIT expressed on the surface of mammalian cells. Furthermore, it was found that the affibody variants could block the interaction between TIGIT and CD155 in an *in vitro* SPR setup, motivating further development as immune checkpoint inhibitors.

## Results

### TIGIT-Specific Affibody Molecules Were Isolated from an *E. coli* Display Library

In order to generate TIGIT-binding affibody molecules, an affibody library with 15 randomized positions, displayed on *Escherichia coli* (*E. coli*), was used (Figure 1A).^38,39^ Each affibody variant in the library was expressed as a fusion protein with an albumin binding domain (ABD). Selection was carried out by four rounds of magnetic-activated cell sorting (MACS), in which the *E. coli* cells were incubated with biotinylated TIGIT (the extracellular domain, ECD), followed by capture with streptavidin immobilized on a solid support. The output from the four MACS rounds was then subjected to two rounds of fluorescence-activated cell sorting (FACS), where the *E. coli* cells were simultaneously stained with fluorescently labeled TIGIT and human serum albumin (HSA). The former to identify target binding, and the latter, through binding to the ABD, was used to determine relative surface expression (Figure 1B). After each selection round, isolated *E. coli* cells were analyzed by flow cytometry for target binding and affibody surface expression to assess the enrichment of the target-binding population (Figure 1C). Throughout the selection campaign, enrichment of the TIGIT-binding population increased from 6.6% after the first MACS round to 73.2% positive clones after the second FACS round, indicating that the selection strategy was successful in generating affibody molecules with affinity toward TIGIT.

**Figure 1:**
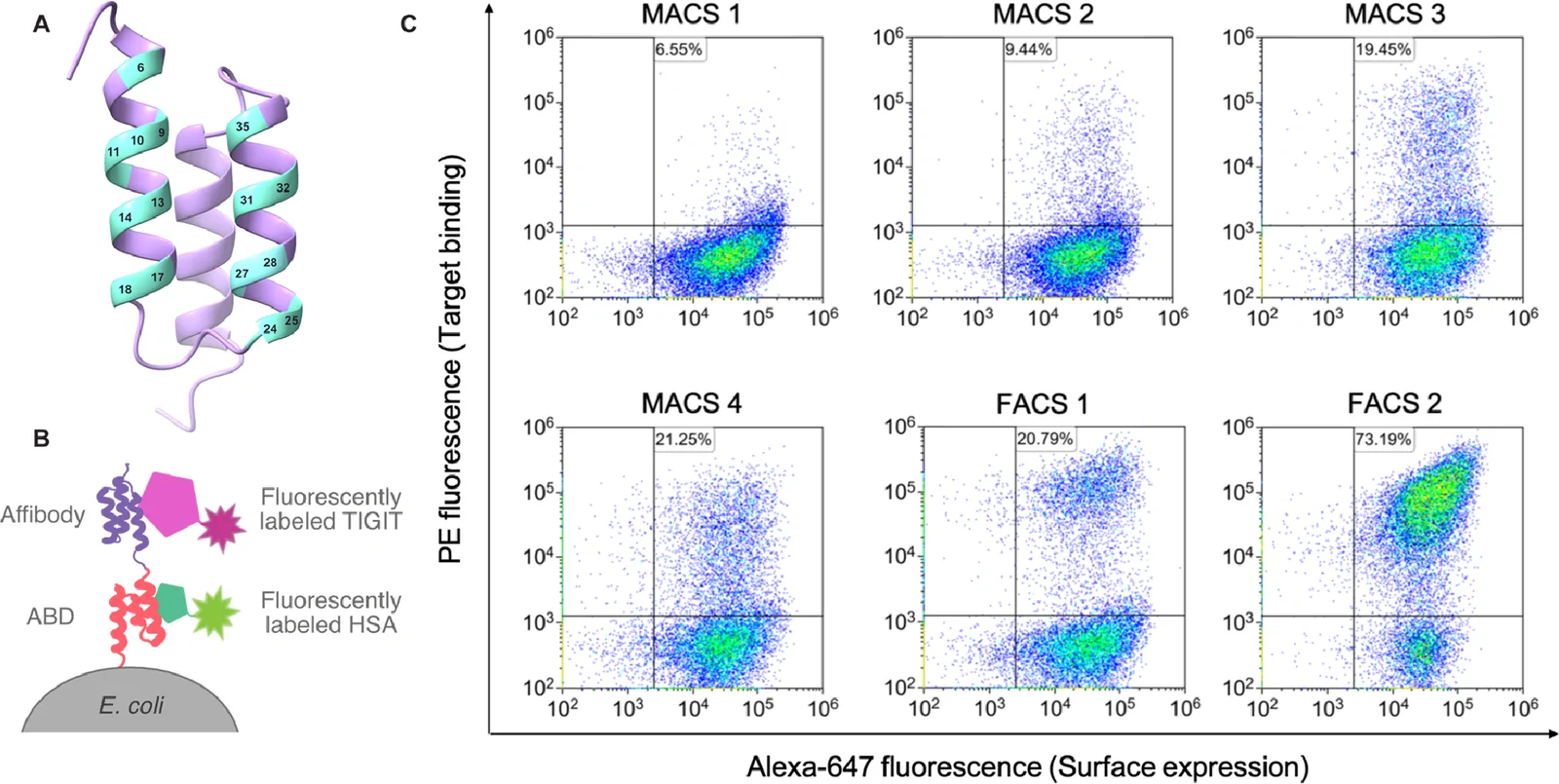
Overview of the selection campaign. (A). The structure of an affibody molecule with 15 randomized positions highlighted in teal. The image was generated in ChimeraX based on the NMR structure of the parental affibody scaffold, the Z-domain (PDB entry 1H0T).^40^ (B). Schematic representation of the *E. coli* display setup. Library members are expressed as a fusion with an ABD on the surface of *E. coli* cells. Staining for target binding with fluorescently labeled TIGIT (the extracellular domain) and relative surface expression level with fluorescently labeled HSA. (C). Analysis of the output after each selection round showed an enrichment of clones binding to TIGIT (upper right quadrant) throughout the six selection rounds, from 6.6% to 73.2%.

From the output after the second round of FACS, 96 *E. coli* colonies were randomly selected. They were stained for TIGIT-binding and relative display level and analyzed by flow cytometry. The DNA sequences of the isolated affibody variants were also determined. The analyses identified 15 unique affibody variants with a desired binding profile (Figure 2A,B). The affibody molecules were recombinantly expressed as soluble fusion proteins with a His_6_-tag and an ABD (affibody-His_6_-ABD), followed by evaluation of target binding by surface plasmon resonance (SPR) analysis with the extracellular domain of TIGIT immobilized on the sensor chip surface. Out of the 15 clones, 13 exhibited detectable affinity for TIGIT (Figure 2C). The clones 2, 6, and 10 (denoted Z_TIGIT:002_, Z_TIGIT:006_, and Z_TIGIT:010_) showing slow off-rates and the most promising binding profiles were selected for further analysis.

**Figure 2:**
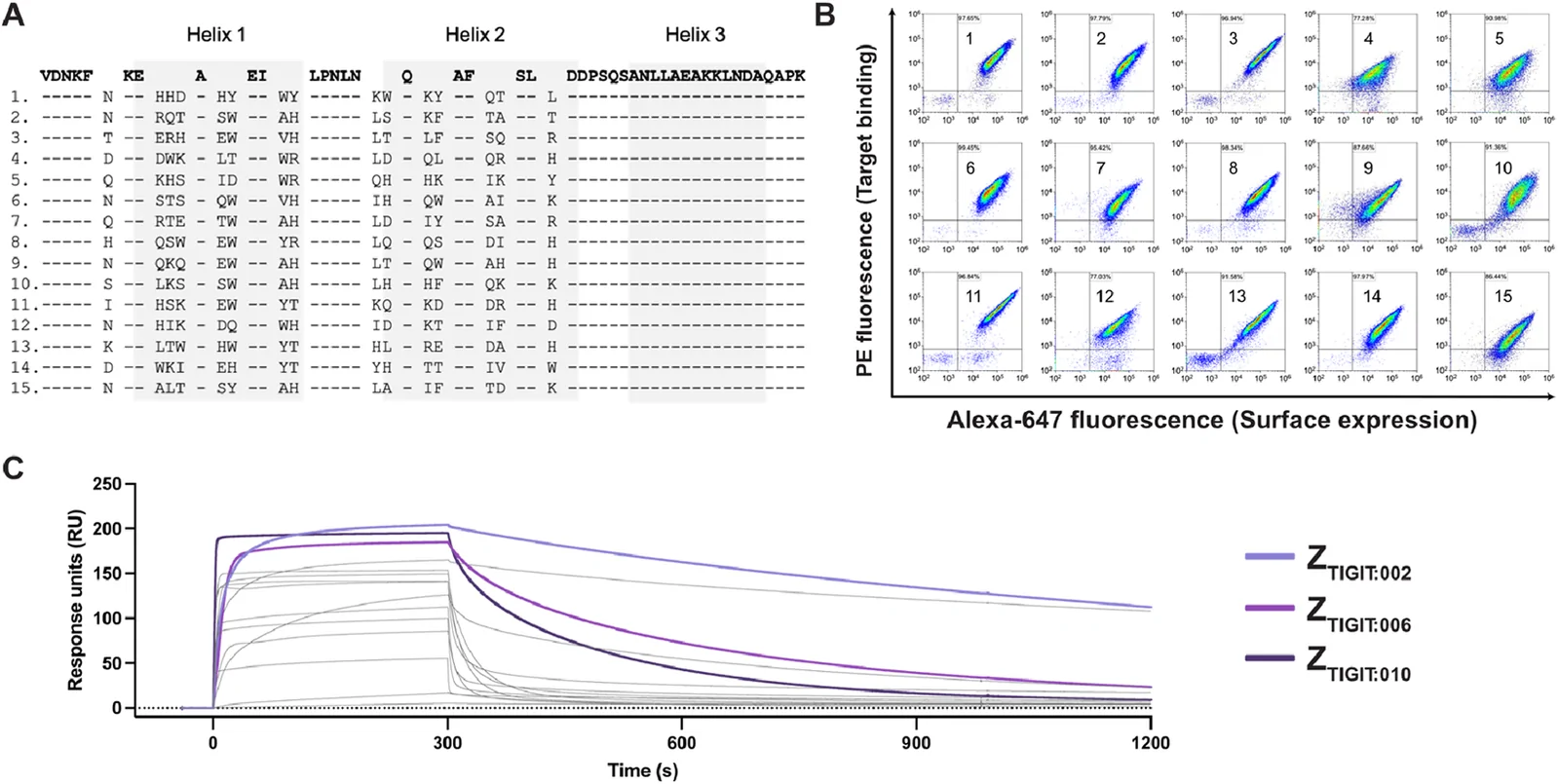
Evaluation of the binding capability of enriched clones after selection. (A). Amino acid sequences of 15 unique variants. The three helices of the affibody molecules are highlighted in gray. Dashes denote amino acids identical to the sequence of the affibody scaffold, in bold at the top. (B). Dot plots obtained by flow cytometry analysis of the 15 affibody molecules. The cells were stained with Alexa647-labeled HSA for determination of surface expression and biotinylated TIGIT (ECD) followed by Phycoerythrin (PE)-labeled streptavidin for identification of target binding. (C). Overlay of sensorgrams obtained after sequential injection of 200 nM of each of the 15 affibody variants over a chip surface with immobilized TIGIT (ECD). Highlighted in purple are the three variants that were selected for further analysis, where Z_TIGIT:002_ corresponds to clone 2, Z_TIGIT:006_ corresponds to clone 6 and Z_TIGIT:010_ corresponds to clone 10 in panels A and B.

### Analysis of Binding Kinetics of the Top Candidates Revealed Low Nanomolar Affinities

The three top candidates, Z_TIGIT:002_, Z_TIGIT:006_ and Z_TIGIT:010_, were produced as monomers with a His_6_-tag in *E. coli* and were purified by immobilized metal-ion affinity chromatography (IMAC). The isolated affibodies were analyzed by SDS-PAGE (Figure 3A), which showed highly pure proteins with the expected molecular weights. Their affinities for TIGIT (ECD) were then determined by SPR to be in the nanomolar range for all three candidates, with Z_TIGIT:006_ exhibiting the strongest affinity (*K*_D_ = 2.5 nM), having the slowest off-rate (Figure 3B, Table 1).

**Figure 3:**
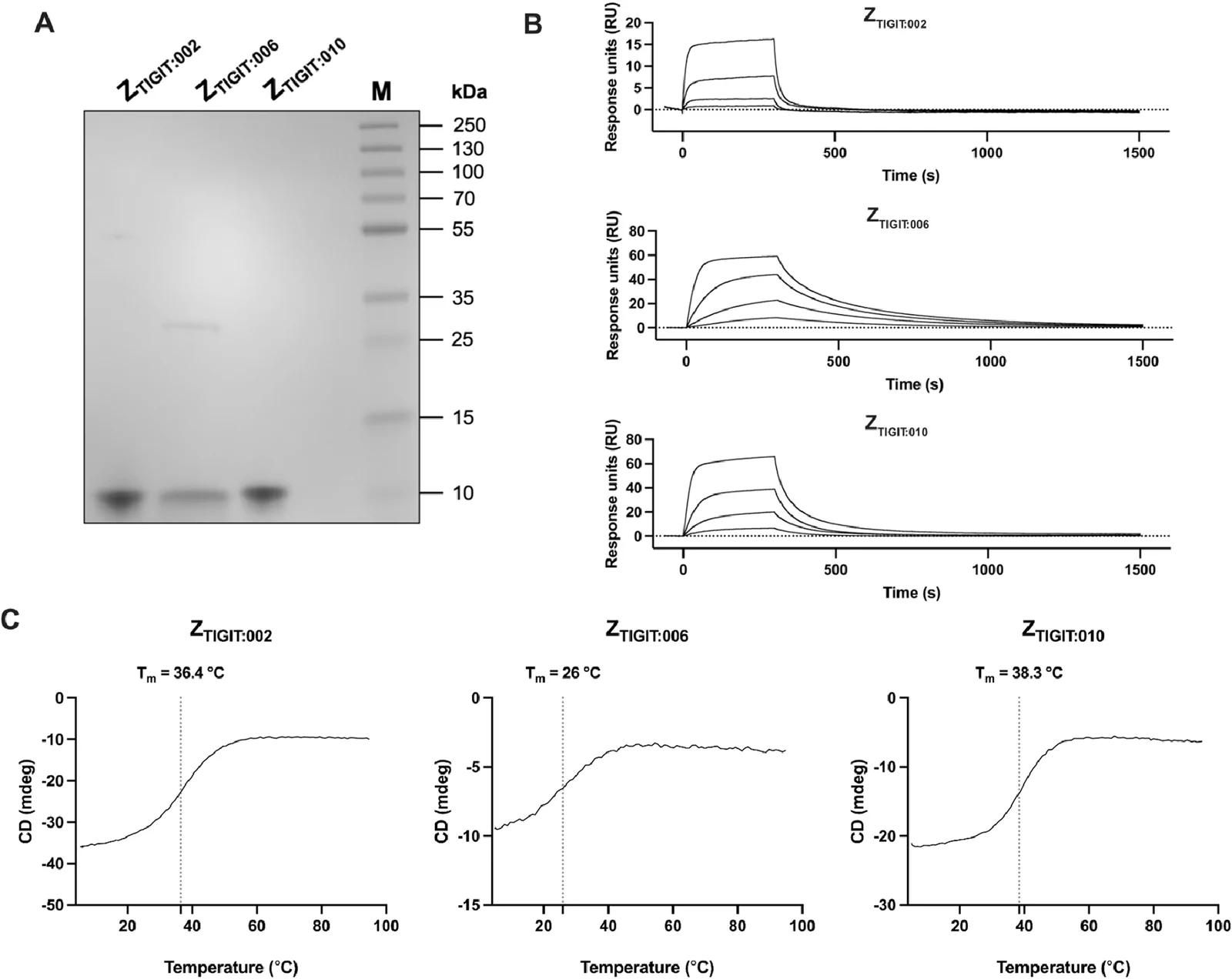
Biochemical analysis of the selected affibody variants. (A). SDS-PAGE analysis of the purified affibodies, revealing highly pure proteins. A protein standard was loaded in lane M with the molecular weights of the standard proteins indicated to the right of the gel (B). Overlay of the sensorgrams obtained after injection of dilution series (18, 6, 2, and 0.7 nM) of each affibody candidate over a surface with immobilized TIGIT (ECD). Each concentration was injected in duplicates. (C). Melting curves for Z_TIGIT:002_, Z_TIGIT:006_, Z_TIGIT:010_, with melting temperatures indicated with the dotted vertical line. The curves were obtained by increasing the temperature from 4 to 99 °C, while measuring the ellipticity at 222 nm.

**Table 1:** Kinetic Parameters for the Affibody/TIGIT Interaction Determined by SPR

Analyte	*K*_D_ (nM)	*k*_a_ (M^–1^s^–1^)	*k*_d_ (s^–1^)
Z_TIGIT:002_	22	4.5 × 10^6^	9.7 × 10^–2^
Z_TIGIT:006_	2.5	3.3 × 10^6^	0.8 × 10^–2^
Z_TIGIT:010_	6.5	2.8 × 10^6^	1.9 × 10^–2^

### Circular Dichroism Analysis Revealed a Characteristic α-Helical Secondary Structure Content of the Top Candidates

To evaluate the secondary structure content and thermal stability of the top candidates, circular dichroism (CD) spectroscopy was used. Variable-wavelength measurements showed spectra with two minima at 208 and 222 nm, indicative of proteins with high α-helical content (Figure S1). The ellipticities were less than expected of a fully folded three-helix bundle such as the original affibody scaffold, indicating that the folding of the helices of the top candidates was less stable than in the original affibody scaffold. The affibodies were then heated from 4 to 99 °C, while recording the CD signal at 222 nm, where α-helices have a highly negative value. The analysis revealed a transition from an α-helical fold to unstructured with melting points between 26.0 and 38.3 °C (Figure 3C). These melting temperatures are low compared to that of the original affibody scaffold, which is around 78 °C.^41^ However, variable-wavelength measurements after thermal denaturation followed by cooling showed spectra that were essentially identical to the spectra recorded before thermal denaturation (Figure S1). This indicated that all three candidates were able to refold after heat-induced denaturation. Moreover, the results supported further engineering of the affibody-variants to increase the melting point for future *in vivo* experiments.

### Affibody Candidates Bind TIGIT on the Surface of Mammalian Cells and Block the Interaction between CD155 and TIGIT in a Concentration-Dependent Manner

To assess the potential of the two candidates with the highest affinity (Z_TIGIT:006_ and Z_TIGIT:010_) to interact with full-length TIGIT, their ability to bind TIGIT on the surface of mammalian cells was evaluated. In order to detect binding, the affibodies were expressed as fusion proteins with an ABD. Dimers of the affibody molecules were also produced to assess whether dimerization could increase the apparent target-affinity and enhance blocking of natural ligands, *e*.*g*., CD155. The ABD-fused constructs were analyzed by SDS-PAGE, showing essentially pure proteins with the expected molecular weights (Figure S2). HEK293T cells, which do not naturally express TIGIT, were transiently transfected with a plasmid encoding full-length TIGIT, followed by staining with either an anti-TIGIT antibody (positive control), Z_TIGIT:006_, Z_TIGIT:010_, or the dimeric form of the affibodies, di-Z_TIGIT:006_ and di-Z_TIGIT:010_. The positive control antibody showed that the transfected cells expressed TIGIT (Figure 4A), which was deemed sufficient for evaluation of the mammalian cell-binding capabilities of the affibodies. Interestingly, two cell populations are observed, with high and very high TIGIT-expression, respectively. In Figure 4B, a schematic overview of the monomeric and dimeric constructs is displayed. Both affibodies displayed binding to the cells, with Z_TIGIT:006_ showing a larger shift in fluorescence (Figure 4C,D). The two cell populations observed with the positive control antibody were also detected, but were less obvious. Staining the cells with the dimeric constructs gave a larger shift in fluorescence compared to the monomeric construct, with a slightly larger shift for di-Z_TIGIT:006_ compared to di-Z_TIGIT:010_. Both cell populations were observed with the dimeric constructs and were more distinct compared to the monomeric constructs. As a specificity control, nontransfected HEK293T cells were stained in the same way (Figure S3). As expected, the positive control antibody did not interact with the cells. For the affibody constructs, the recorded histograms essentially overlapped for nonstained cells, cells stained with secondary reagent only, and cells stained with an affibody construct and secondary reagent, suggesting that the affibody constructs did not interact nonspecifically with HEK293T cells.

**Figure 4:**
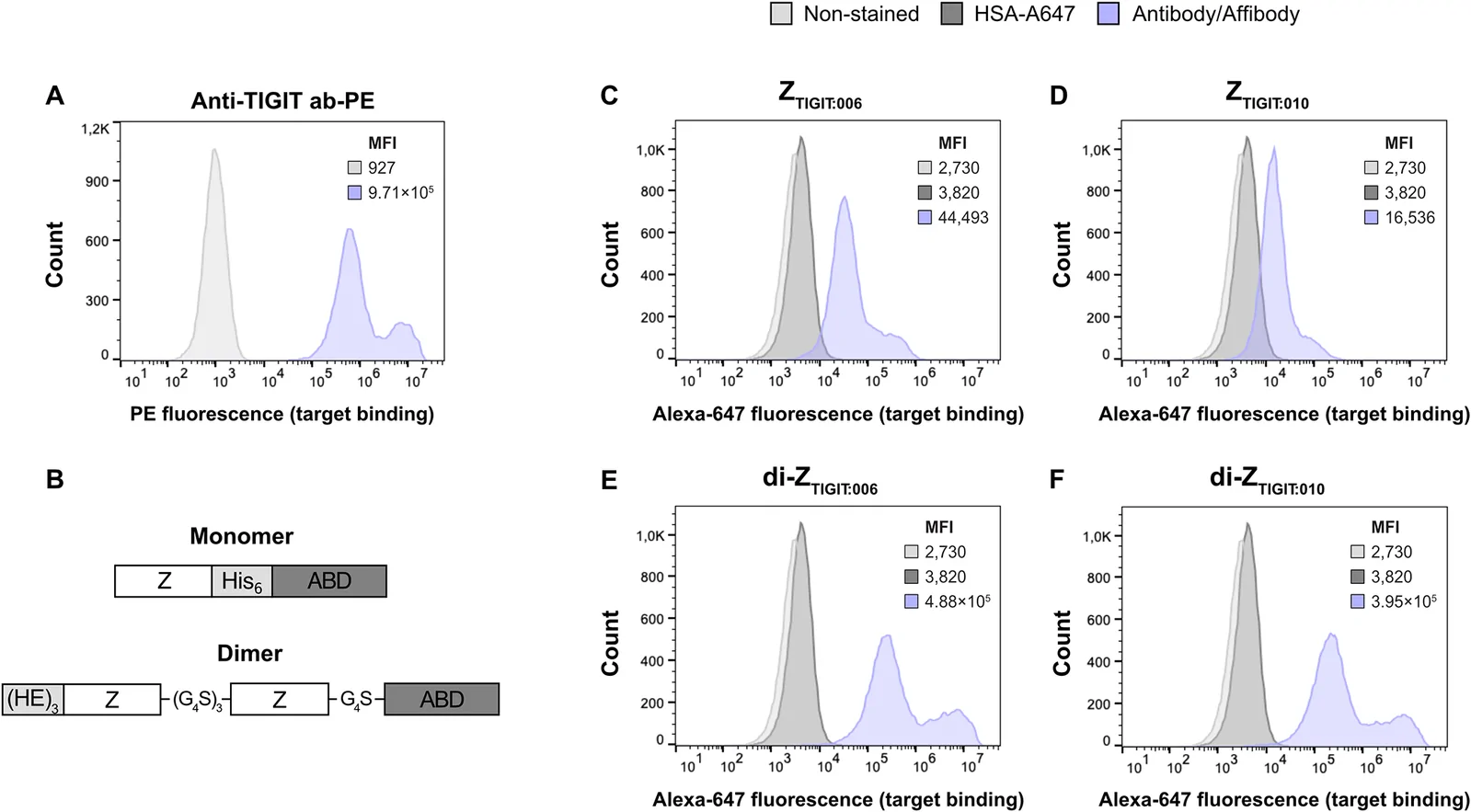
Flow cytometry analysis evaluating the cell binding capabilities of affibody candidates. (A). HEK293T cells expressing full-length TIGIT were stained with an anti-TIGIT antibody conjugated to PE (violet) and nonstained cells (gray). (B). Schematic representation of the monomeric and dimeric constructs with Z indicating affibody molecules. The cysteines in the dimeric constructs were alkylated for the experiment. (C, D). HEK293T cells expressing TIGIT stained with Z_TIGIT:006_ or Z_TIGIT:010_ (100 nM) followed by HSA labeled with Alexa-647 (HSA-A647) (violet) as secondary reagent. As controls, nonstained cells (light gray) and cells stained with only the secondary reagent (dark gray) were analyzed. (E, F). HEK293T cells expressing TIGIT stained with di-Z_TIGIT:006_ or di-Z_TIGIT:010_ (100 nM, violet) together with HSA-A647 as secondary reagent or with only the secondary reagent (dark gray) and nonstained cells (light gray). Each analysis was performed in duplicates, which essentially overlapped. For clarity, only one of the recorded histograms is presented in the figure. Mean fluorescence intensities (MFI) were calculated for the major peak for each histogram and are displayed in the panels.

Next, an SPR-assay was used to evaluate the ability of the affibody molecules to block the interaction between TIGIT and CD155. First, a concentration series of the affibody constructs was preincubated with TIGIT (ECD) for 3 h on ice to allow a complex to form, followed by injection over a sensor chip with immobilized CD155. If the affibody bound to TIGIT (ECD) with an orientation that prevented the TIGIT/CD155 interaction, no binding would be detected. Consequently, a nonantagonistic interaction between the affibody and TIGIT (ECD) would allow the TIGIT/CD155 complex to form, and a signal to be detected (Figure 5A). In this particular setup, the results show that Z_TIGIT:006_, could completely block the interaction at a concentration of 600 nM affibody to 50 nM TIGIT (Figure 5B). Z_TIGIT:010_ was more potent and showed complete blocking at 200 nM affibody to 50 nM TIGIT (Figure 5B). The dimeric constructs were slightly more potent than the monomeric constructs, and complete blocking by di-Z_TIGIT:006_ and di-Z_TIGIT:010_ was achieved already at 200 and 67 nM, respectively.

**Figure 5:**
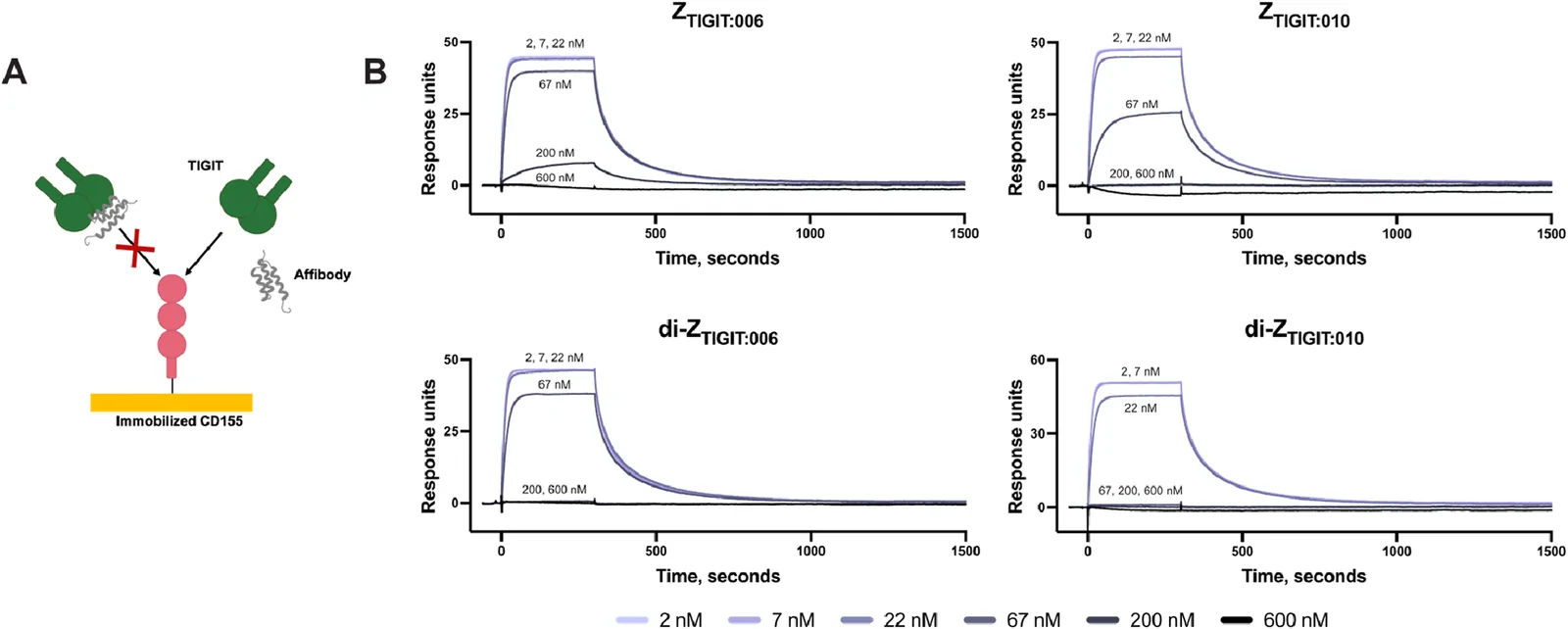
Evaluation of inhibition of the TIGIT/CD155 interaction. (A). Schematic representation of the experimental setup. (B). Overlay of SPR sensorgrams displaying a concentration-dependent inhibition of the TIGIT/CD155 interaction. The affibody construct used during preincubation with TIGIT (ECD) is indicated above each sensorgram, and the numbers above each curve indicate the affibody concentration (light violet to black), incubated with a constant TIGIT concentration of 50 nM prior to injection over a sensor chip with immobilized CD155.

### Leveraging General Language Models to Improve the Thermostability

In an effort to improve the thermostability of the top candidates, Z_TIGIT:006_, and Z_TIGIT:010_, were subjected to an *in silico* evolution using the ESM-1v ensemble of five language models.^42^ The output suggested one substitution for Z_TIGIT:006_, and four substitutions for Z_TIGIT:010_ (Figure 6A). All suggested substitutions were in the variable positions of the affibody, which could affect the affinity for TIGIT. However, since the affibodies’ paratopes are unknown, all the single mutant variants suggested by the ensemble were produced and characterized. All single mutants showed highly α-helical secondary structure content similar to the original variants (Figure S1). Out of the five mutants, three retained a detectable affinity for TIGIT. All three had slightly higher melting temperatures than their respective original affibody (Figure 6B), where the highest was observed for Z_TIGIT:010_H27L_ (42.7 °C), which was 4.4 °C higher than the original variant (Z_TIGIT:010_).

**Figure 6:**
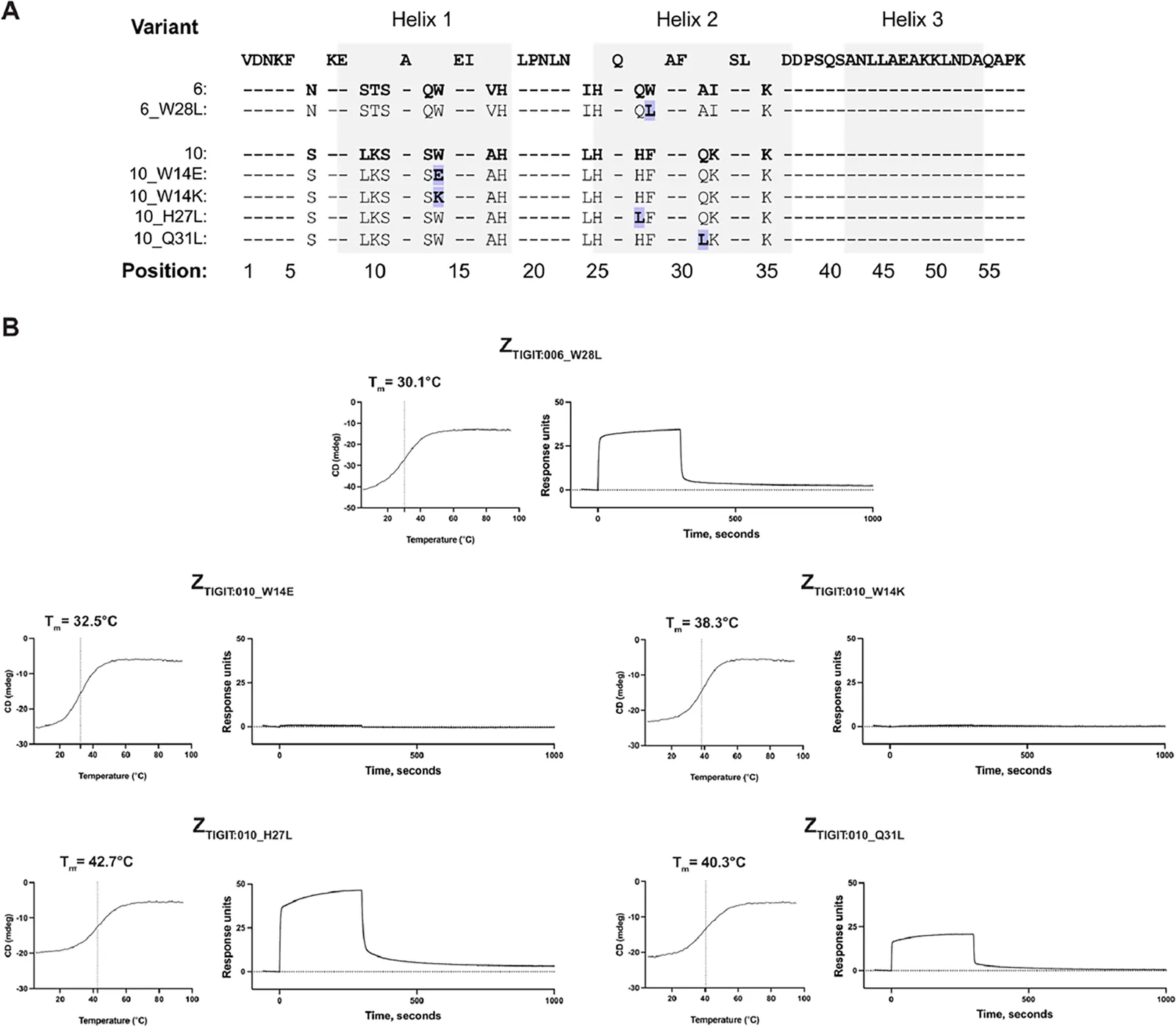
Characterization of single mutant variants. (A). Sequences of variants suggested by the general language models with amino acid mutation highlighted in violet. (B). Melting curves determined by CD where θ_222_ was recorded as a function of increased temperature. The variants were injected (200 nM) over a chip with immobilized TIGIT and the recorded sensorgrams are displayed.

Next, the kinetic constants and the equilibrium dissociation constants of Z_TIGIT:006_W28L_, Z_TIGIT:010_H27L_ and Z_TIGIT:010_Q31L_, were determined by SPR (Figure S4). The affinities for all three variants were weaker compared to their original counterparts, however, with *K*_D_-values still in the nanomolar range (Table 2).

**Table 2:** Kinetic Parameters Determined by SPR for the Three Single Mutant Variants That Displayed Binding to TIGIT

Analyte	*K*_D_ (nM)	*k*_a_ (M^–1^s^–1^)	*k*_d_ (s^–1^)
Z_TIGIT:006_W28L_	37	2.4 × 10^4^	8.8 × 10^–4^
Z_TIGIT:010_H27L_	12	1.1 × 10^5^	1.3 × 10^–3^
Z_TIGIT:010_Q31L_	14	5.6 × 10^4^	7.8 × 10^–4^

## Discussion

Immune checkpoint therapies (ICT) have shown great success in clinical practice during the past 15 years. However, the current treatments are associated with several severe side effects, and only a limited subset of patients respond to currently available treatments.^43,44^ Hence, investigation of novel targets and targeting molecules with other characteristics is motivated. Therefore, we have in this study selected affibody molecules to the T cell immunoglobulin and ITIM domain (TIGIT) receptor from a large combinatorial library displayed on *E. coli*, followed by engineering for enhanced thermostability by capitalizing on mutations suggested by an ensemble of general language models.^45,46^

The selections from the affibody library resulted in anti-TIGIT affibody molecules with low-nanomolar equilibrium dissociation constants (*K*_D_ values). Their binding capabilities were evaluated by SPR and by flow cytometry using TIGIT-expressing cells. Furthermore, their ability to block the interaction between TIGIT and its natural ligand CD155 was evaluated in an SPR assay. The two best-performing variants bound to TIGIT expressed on the surface of mammalian cells and could block the interaction between TIGIT and CD155. Dimeric versions of the affibody molecules showed blocking capabilities at lower concentrations compared to their monomeric counterparts, suggesting that this format is the most promising for further development as a checkpoint inhibitor, possibly in combination with other ICTs such as mAb targeting PD-1, similar to the clinical study by Hsiehchen et al.^47^ However, the results from the blocking experiment in SPR can only be interpreted as a qualitative measurement, to show that blocking is possible. The expression of TIGIT and CD155 in animal models of cancer and cancer patients will be different, and careful optimization of the format and dosing has to be performed in future studies with these affibody molecules. Moreover, although Z_TIGIT:006_ displayed a stronger affinity than Z_TIGIT:010_ in the SPR analysis, the latter more effectively blocked the TIGIT/CD155 interaction in the SPR experiment. This could be due to differences in epitope binding orientation, *i*.*e*., that Z_TIGIT:010_ more efficiently covered the binding site on TIGIT for CD155. In the current study, TIGIT blockade was demonstrated biochemically using SPR. The ability of these affibody molecules to restore or enhance T cell and/or NK cell function remains to be established.

The *K*_D_ values of the interactions between TIGIT and the generated affibody molecules Z_TIGIT:006_ and Z_TIGIT:010_ were 2.5 and 6.5 nM, respectively. While affibodies with picomolar affinity for their target have been described,^48^ selection campaigns from naïve affibody libraries typically yield variants in the nanomolar range, similar to the results in this study. Affibody molecules can be expressed as head-to-tail multimers, and in many cases this yields a stronger functional affinity to the target. This avidity effect was observed for the dimeric constructs di-Z_TIGIT:006_ and di-Z_TIGIT:010_, which exhibited markedly slower off-rates in an SPR analysis (Figure S5).

It has previously been shown that TIGIT expression on tumor-infiltrating lymphocytes (TILs) correlates with the objective response to treatment with immune checkpoint inhibitors targeting TIGIT in patients.^49^ Therefore, it is also of importance to develop efficient probes for quantifying TIGIT expression in tumors. Affibody molecules have previously been radiolabeled and have been found to be excellent probes for radionuclide molecular imaging using PET and SPECT.^50^ An example is affibodies targeting HER2, which have been shown to give excellent contrast images both in mouse models and in breast cancer patients.^48^ Due to their small size, affibody molecules are rapidly cleared by the kidneys, allowing imaging within a few hours postinjection (typically 1–4 h),^48^ whereas antibodies such as ^89^Zr-trastuzumab, require waiting 4–5 days before imaging to achieve a good contrast.^51^ Recently, a study describing the selection of nanobodies for imaging of TIGIT expression was published.^29^ The authors showed that the nanobodies enabled high-contrast *in vivo* imaging of TIGIT expression as early as 1 h postinjection. One benefit of being able to image TIGIT expression *in vivo* is that it enables noninvasive visualization and quantification of TIGIT-expressing TILs, which can provide a basis for selecting the treatment most likely to benefit the patient.^49^

To determine the therapeutic effect of the generated affibody molecules, their efficacy should, in future studies, be evaluated *in vivo*. One potential strategy could be to investigate the effect on xenografted tumors in mice with a humanized immune system. However, as a first proof-of-concept to demonstrate tumor accumulation of the affibody molecules, a xenografted tumor expressing TIGIT could be used as a proxy, followed by PET or SPECT imaging, similarly to the assay performed by Zeven et al.^29^ It would also shed light on the feasibility of imaging TIGIT expression *in vivo* by the affibodies. The affinities of the top two affibodies (2.5 and 6.5 nM) are comparable to that of the developed nanobodies (0.1 nM (mTIGIT) and 2 nM (hTIGIT)). The molecular weight of an affibody is roughly half the size of a nanobody, which should provide better extravasation and tumor accumulation.^32,52^ Moreover, in the same nanobody-study a scFv fragment was generated from the clinically validated mAb vibostolimab targeting TIGIT.^53^ The scFv fragment displayed a *K*_D_ value of 1 nM and a melting temperature of 69 °C. Both nanobodies and scFvs utilize loops for interacting with their target in contrast to affibody molecules, which uses a relatively flat surface, which could be superior for interacting with TIGIT, and hence the stronger affinity. However, using affinity maturation, affibody molecules have been developed against other targets, such as HER2, with *K*_D_ values down to 22 pM,^48^ suggesting the possibility of further engineering of Z_TIGIT:006_ and Z_TIGIT:010_ to find variants with even better equilibrium dissociation constants.

There is an ongoing debate on whether Fc-interaction enhances the therapeutic effect of anti-TIGIT immune checkpoint inhibitors.^54–56^ Affibody molecules do not have an intrinsic ability to interact with Fc-receptors, however, multiple studies have shown that they have excellent capabilities as modules in fusion proteins with different architecture. Affibody molecules targeting syncytial virus surface protein G and *Thermus aquaticus* DNA polymerase have, for example, been expressed as fusion proteins to an IgG-derived Fc-domain, creating constructs that combine the functionality of both parts.^35,57^ This makes Fc-fusion a potential strategy for further development of affibody-based anti-TIGIT ICIs. Conversely, there are research programs on anti-TIGIT antibodies with the Fc-region silenced, such as domvanalimab, currently undergoing several phase III clinical trials (*e*.*g*., NCT05502237 and NCT05568095).^22^ A purpose of silencing the Fc-domain is to prevent antibody-dependent cellular cytotoxicity (ADCC)-mediated depletion of peripheral immune cells expressing TIGIT, which is undesirable since these cells are required for an efficient antitumor response.

To be suitable for *in vivo* experiments a melting temperature of at least 45 °C is desirable to ensure complete folding at physiological temperature. Z_TIGIT:006_ and Z_TIGIT:010_ both had lower melting temperatures, which necessitate further engineering before they are suitable for *in vivo* experiments. In an effort to improve the thermostability of the top candidates, the potential of using machine learning algorithms was explored. Recent advances in artificial intelligence (AI) and machine learning have enabled the development of powerful tools in the field of biotechnology, for example in protein science. Beyond structure prediction, enabled by *e*.*g*., AlphaFold and Rosetta,^58–60^ machine learning algorithms is increasingly being applied to guide other properties in protein design, for example by suggesting mutations that may increase binding affinity or stability of the protein.

We selected the ESM-1v ensemble of five language models developed by Hie et al. for suggestions for amino acid substitutions.^42^ The ensemble was originally developed for engineering of antibodies by suggesting evolutionarily plausible mutations. In the study by Hie et al., the models in the ensemble suggested multiple variants that were shown to have an increased affinity for their target, often accompanied by an increased melting temperature. The ensemble of models has also been employed for the evolution of affibody molecules targeting CD69, where it suggested variants that displayed a retained affinity accompanied by an increased melting temperature of up to 9 °C.^61^ For Z_TIGIT:006_ and Z_TIGIT:010_ the models of the ensemble suggested five single mutant variants based on the original affibodies. Only three of them retained binding to TIGIT, and these three had a 2- to 15-fold decreased affinity compared to the original variants, indicating that the ensemble was not optimal for *in silico* evolution of Z_TIGIT:006_ and Z_TIGIT:010_ (Figure S4).

Machine learning algorithms for engineering protein properties are currently evolving quickly and there are several tools available. Some examples focused on enhancing thermal stabilities, using either sequence data or structural data or a combination of the two, includes THPLM,^62^ DDMut^63^ and mutDDG-SSM.^64^ However, since these methods require experimentally determined structures, they cannot be applied to the affibody molecules developed in this study yet, due the lack of structural data.

Another approach to identify variants with stronger affinity could be an affinity maturation campaign, where parts of the lead binder is rerandomized to create a new library followed by selection under stringent conditions. In the study by Olsson et al. on affibody molecules targeting CD69, the affinity maturation campaign led to the identification of a variant with 35-fold stronger affinity, however, with a decrease of *T*_m_ from 59 to 44 °C.

In conclusion, we have developed two affibody molecules with strong affinities to TIGIT that can block the interaction between TIGIT and CD155 *in vitro*, which show great promise for future applications for *in vivo* imaging of TIGIT expression and immune checkpoint therapy.

## Materials and Methods

### General

Materials were obtained from Merck (Darmstads, Germany), Thermo Fischer Scientific (Waltham, MA, USA), or VWR (Radnor, PA, USA) unless stated otherwise.

### Affibody Library and Selection of TIGIT Binders

To generate affibody binders against TIGIT, a previously constructed *E. coli* display library consisting of 1.57 × 10^11^ colony-forming units was subjected to six rounds of selections.^38,39^ The selection was performed using recombinant biotinylated TIGIT (ECD) (cat. no. TIT-H82E6, ACROBiosystems, Newark, DE, USA) and the selection strategy was adapted from Parks et al.^38^

The library was expanded by inoculating an aliquot of 1 × 10^11^ cells (approximately a one-fold coverage of the library) of the library stock in 1.25 L of Lysogeny Broth (LB) media and incubating the cultures at 37 °C overnight. The next day, 1 × 10^11^ cells were collected from the overnight culture and were diluted in 1.25 L LB medium and grown at 37 °C until the optical density at 600 nm (OD_600_) reached 0.6–0.8. The culture was induced with arabinose to a final concentration of 0.6% and incubated at 25 °C overnight. The following day, 1 × 10^11^ cells were transferred to a tube and PBSP (phosphate-buffered saline (PBS) supplemented with 0.1% Pluronic F108 NF surfactant (BASF Corporation, Florham Park, NJ, USA)) was added to a final volume of 50 mL and centrifuged for 10 min at 4000*g* at 4 °C. The cells were washed three times with PBSP and were then ready for MACS or FACS.

For MACS, a negative selection step was performed where the cells were incubated with Dynabeads MyOne Streptavidin C1 (Invitrogen, Waltham, MA, USA) for 30 min at room temperature (RT). Selection beads were prepared by incubation with 50 nM TIGIT for 1 h at RT. After the negative selection, remaining cells were incubated with the selection beads for 2 h at RT, followed by washing three times with PBSP. Selection beads with remaining cells were diluted in 50 mL LB media and incubated at 37 °C overnight. From the overnight culture glycerol stocks were prepared and a part of the culture was diluted and induced, as described above, for flow cytometry analysis. Three additional MACS rounds were performed similarly, except that the negative selection was omitted.

After the four MACS rounds, the library was subjected to two rounds of FACS, using an Cytoflex SRT (Beckman Coulter, Brea, CA, USA) machine. The *E. coli* library was stained for surface expression with human serum albumin conjugated to Alexa Fluor 647 (HSA-A647) and for target binding using biotinylated TIGIT followed by streptavidin conjugated to phycoerythrin (SAPE). The top 1% of the target binding population was collected. Target concentration was decreased from 30 nM in the first FACS round to 15 nM in the second. For the two FACS rounds, an off-rate selection step was included to enrich for binders with slower dissociation rates. The off-rate selection steps consisted of an additional 1.5 or 3 h incubation in PBSP at 37 °C with 3 or 6 washes, respectively, prior to sorting.

### Selection Output and Single Candidate Screening by Flow Cytometry

Analysis of enrichment of TIGIT binders was performed using a CytoFLEX S flow cytometer (Beckman Coulter). Cell cultures were grown overnight in LB media supplemented with 100 mg/L carbenicillin. A 10 μL aliquot of the overnight culture was washed twice with PBSP followed by incubation with biotinylated TIGIT (ECD, 30 nM) for 45 min at RT. After one wash with PBSP the cells were simultaneously stained with HSA-A647 and SAPE, in a total volume of 100 μL, for 30 min on ice. After washing with PBSP the cells were resuspended in 200 μL PBSP and analyzed in the flow cytometer with 20,000 events recorded per sample. The same protocol was followed for analysis of single clones (Figure 2B).

### Subcloning of Affibody Variants into Different Production Vectors

Genes encoding 15 affibody variants were subcloned to the pET26b+ expression vector (Novagen, Darmstadt, Germany) to yield constructs with the format: affibody-His_6_-ABD. The ABD used was (ABD_035_).^65^ Dimeric variants of Z_TIGIT:006_ and Z_TIGIT:010_ with ABD_035_ was generated as well. Monomeric variants without the ABD were constructed for Z_TIGIT:002_, Z_TIGIT:006_ and Z_TIGIT:010_, where the sequences were subcloned into the pET45b+ vector by In-fusion cloning (TaKaRa Bio, Shiga, Japan). All sequences were confirmed by Sanger sequencing.

### Generation of Single Mutant Variants Suggested by the Ensemble of General Language Models

Single mutant variants (SMVs) were generated using a ensemble of general language models developed by Hie et al.^42^ with an α-value of 0.8. Gene fragments encoding the SMVs were synthesized by Twist Bioscience (San Francisco, CA, USA) followed by subcloning into the pET45b+ expression vector by In-fusion cloning. The DNA sequences of all resulting plasmids were confirmed by Sanger sequencing.

### Production and Purification of Affibody Molecules

The production and purification of the affibody molecules were performed essentially as previously described.^66^ Briefly, the proteins were expressed in *E. coli* BL21 (DE3) Star (New England Biolabs, Ipswich, MA, USA) in 100 mL TSB+Y medium (30 g/L Tryptic soy broth, 5 g/L Yeast extract), supplemented with either 50 mg/L kanamycin or 100 mg/L carbenicillin. The cultures were grown at 37 °C until OD_600_ reached 0.5–1.0, after which protein expression was induced with 1 mM isopropyl β-d-1-thiogalactopyranoside (IPTG) (Appolo Scientific, Stockport, U.K.). The cultures were then incubated at 25 °C overnight, and cells were collected by centrifugation.

For constructs including the ABD, the fusion proteins were purified by affinity chromatography with HSA as immobilized ligand.^66^ Elution from the column was carried out with 0.5 M acetic acid (pH 2.8), followed by lyophilization and storage at −20 °C.

Constructs lacking the ABD were purified by immobilized metal-ion affinity chromatography (IMAC) on a HisPur Cobalt Resin (Thermo Fisher Scientific) under native conditions, according to the manufacturer’s protocol. PBS with 30 mM imidazole was used as the binding and column wash buffer and PBS with 300 mM imidazole was used for elution. The eluted proteins were buffer-exchanged to PBS by dialysis using a 3.5 MWCO Slide-a-Lyser cassette (Thermo Fisher Scientific).

Protein concentrations were determined by a BCA protein assay kit (Thermo Fisher Scientific) according to the manufacturer’s instructions.

### Surface Plasmon Resonance

The affinities of the produced affibody candidates were determined by surface plasmon resonance (SPR) using a Biacore 8K (Cytiva, Uppsala, Sweden). The analysis was performed with PBS supplemented with 0.1% Tween-20, pH 7.4, PBST, as running buffer (30 μL/min). The extracellular domain of human TIGIT (cat. no. TIT-H82F1, ACROBiosystems) was immobilized on a CM5 sensor chip (Cytiva) at pH 4.0. using an amine coupling kit. A reference flow cell was created by activation and deactivation and was subtracted from all sensorgrams. The chip surface was regenerated by injection of HCl (10 mM) for 30 s. Kinetic constants were derived from the sensorgrams using the Biacore evaluation software.

Blocking of the interaction between CD155 and TIGIT by the affibodies was assessed using a Biacore T200 instrument (Cytiva). CD155 (cat. no. CD5-H82E3, ACRObiosystems) was immobilized on the surface of a CM5 sensor chip at pH 4.5 using an amine coupling kit. A reference flow cell was prepared by activation and deactivation and was subtracted from all sensorgrams. Mixtures of TIGIT (ECD) (50 nM) and varying concentrations of affibody (600, 200, 67, 22, 7, and 2 nM) was prepared in PBST and incubated for 3 h on ice. The mixtures were injected over the chip. PBST was used as running buffer at a flow rate of 30 μL/min.

### Circular Dichroism Spectroscopy

The secondary structure content and thermal stability were analyzed by circular dichroism (CD) spectroscopy. The affibodies were diluted to a concentration of 0.2 mg/mL in PBS followed by measurement of the ellipticity across wavelengths from 260 to 195 nm using a Chirascan spectropolarimeter (Applied Photophysics, Leatherhead, U.K.) at RT. Thermal unfolding was evaluated by monitoring the ellipticity at 222 nm while heating the samples from 4 to 99 °C at a rate of 1 °C/min. Following thermal denaturation, the ellipticity at wavelengths between 260 and 195 nm was measured to assess the ability of the affibodies to refold.

### Generation of a TIGIT-Expressing Cell Line and Evaluation of Cell Binding of the Affibodies

A gene encoding the full-length TIGIT gene, including a CD33 signal peptide was synthesized by Thermo Fisher Scientific. The gene was flanked by restriction enzyme sites recognized by NheI and AscI. It was subcloned into the plasmid pKTH16 ^67^ by NheI and AscI restriction enzymes (New England Biolabs).

The HEK293T cell line was obtained from ATCC (Manassas, VA, USA). It was cultivated in DMEM medium supplemented with 10% FBS, 1% penicillin-streptomycin and 2 mM l-Glutamate at 37 °C, 5% CO_2_. HEK293T cells (3 × 10^6^) were seeded into a 10 cm dish and were allowed to attach and grow for 2 d. On the third day, the medium was replaced with antibiotic-free medium prior to transfection. PEI Max (54 μg) (Polysciences, Warrington, PA, USA) was mixed with DNA (18 μg) and incubated for 15 min at room temperature. The mixture was added dropwise to the cells, after which the cells were incubated for 24 h. Transfected and nontransfected control cells were harvested and washed once with PBS supplemented with 1% BSA (PBSB). Then 2 × 10^5^ cells were incubated for 30 min with an anti-TIGIT antibody coupled to PE (Thermo Fisher Scientific, cat. no. 12–9500–42) according to the manufacturer’s instructions, or one of the affibody constructs (100 nM) or PBSB, in duplicates. The cells were washed twice with PBSB, and the cells incubated with the affibody constructs were stained with HSA conjugated with Alexa Fluor 647 (313 nM), which bound to the ABD in the constructs. The cells were washed once with PBSB and were analyzed by a CytoFLEX S flow cytometer. Gating with the FSC-A/SSC-A signals separated cells from debris, and was followed by gating for single cells with the FSC-H/FSC-A signals (Figure S6) before recording 20,000 events per sample.

## Supplementary Material



## Notes

CD spectroscopy spectra of the original and mutated variants (Figure S1); SDS-PAGE of the purified constructs (Figure S2); flow cytometry analysis of the TIGIT-negative cell line (Figure S3); SPR sensorgrams of the mutated variants (Figure S4); SPR sensorgrams comparing the monomeric and dimeric constructs (Figure S5); gating strategy for flow cytometry (Figure S6) (PDF)

## ∥

NRG Pallas BV, Medical Isotope Production, Petten 1755 ZG, Noord Holland, NL
