# Supplementary material for: Selection of Anti-TIGIT Affibody Molecules Blocking the Interaction between TIGIT and CD155
Source: ACS Omega. 2026 Oct 22;11(43):66399–410. doi: 10.1021/acsomega.6c05539 (PMC13737976; doi:10.1021/acsomega.6c05539)
Supplement: Supplementary file 1 [file ao6c05539_si_001.pdf]

Supplementary data

Selection of Anti-TIGIT Affibody Molecules Blocking the Interaction between TIGIT and CD155

Linda Ehrenborg[1,†], Javad Garousi[1,†,a], Jie Zhang[1], Hans Grönlund[2], Torbjörn Gräslund[1,2*]

[1]Department of Protein Science, KTH Royal Institute of Technology, Roslagstullsbacken 21, 106 91 Stockholm, Sweden.
[2]Therapeutic Immune Design, Department of Clinical Neuroscience, Karolinska Institutet, Center for Molecular Medicine, Visionsgatan 18, L8, 171 77, Stockholm, Sweden

†These authors contributed equally to this work
[*]Corresponding author: torbjorn@kth.se; Tel.: +46-(0)8-790-9627

[a] Current affiliation: NRG Pallas BV, Medical Isotope Production, Petten, 1755 ZG, Noord Holland, NL

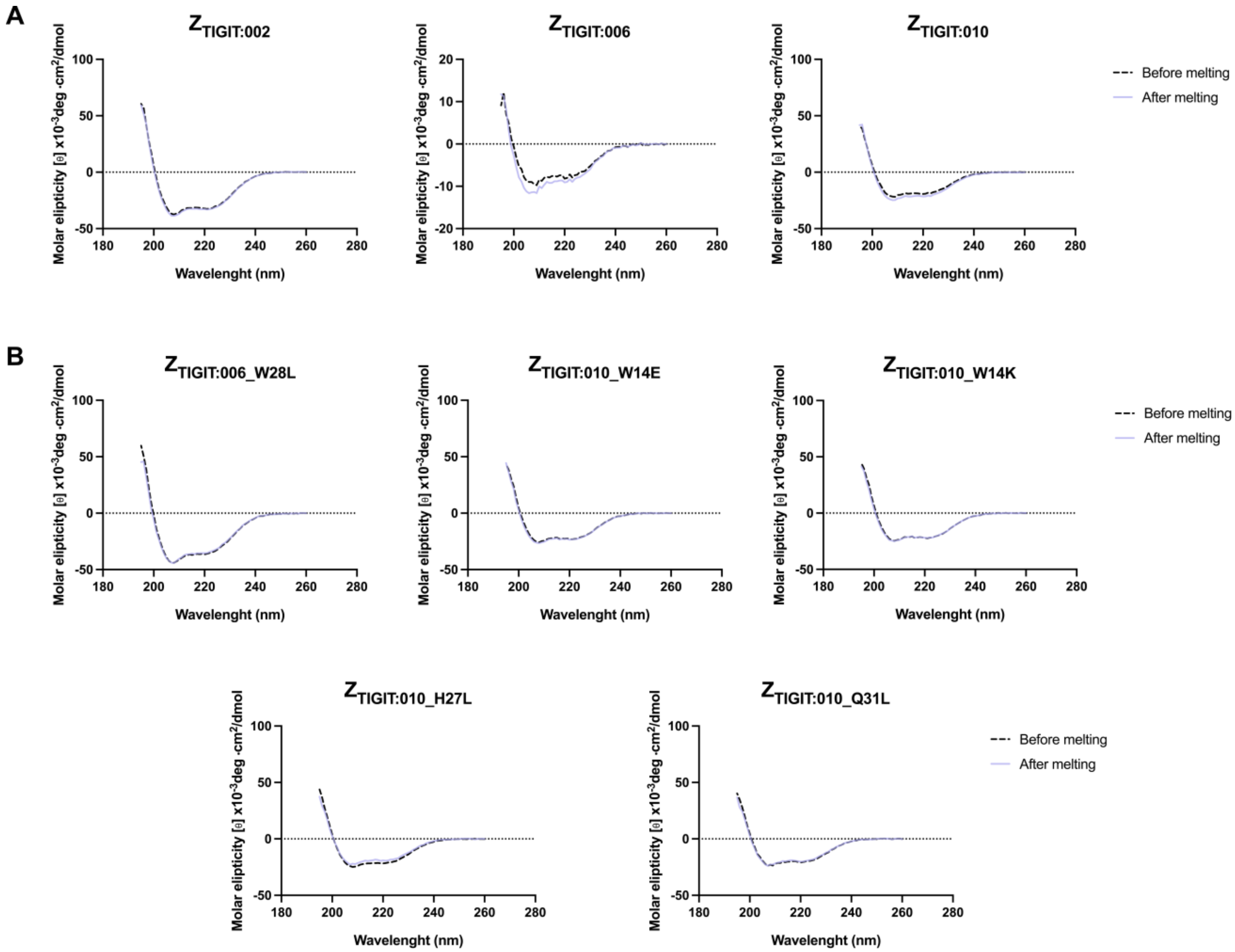

Figure S1. Analysis of the secondary structure content by circular dichroism (CD) spectroscopy. CD-spectra from 260 to 195 nm were collected at an affibody concentration of 0.2 mg/mL in PBS at room temperature. **A.** corresponds to variants identified after the original selection campaign, and **B.** corresponds to variants suggested by the assembly of general language models.

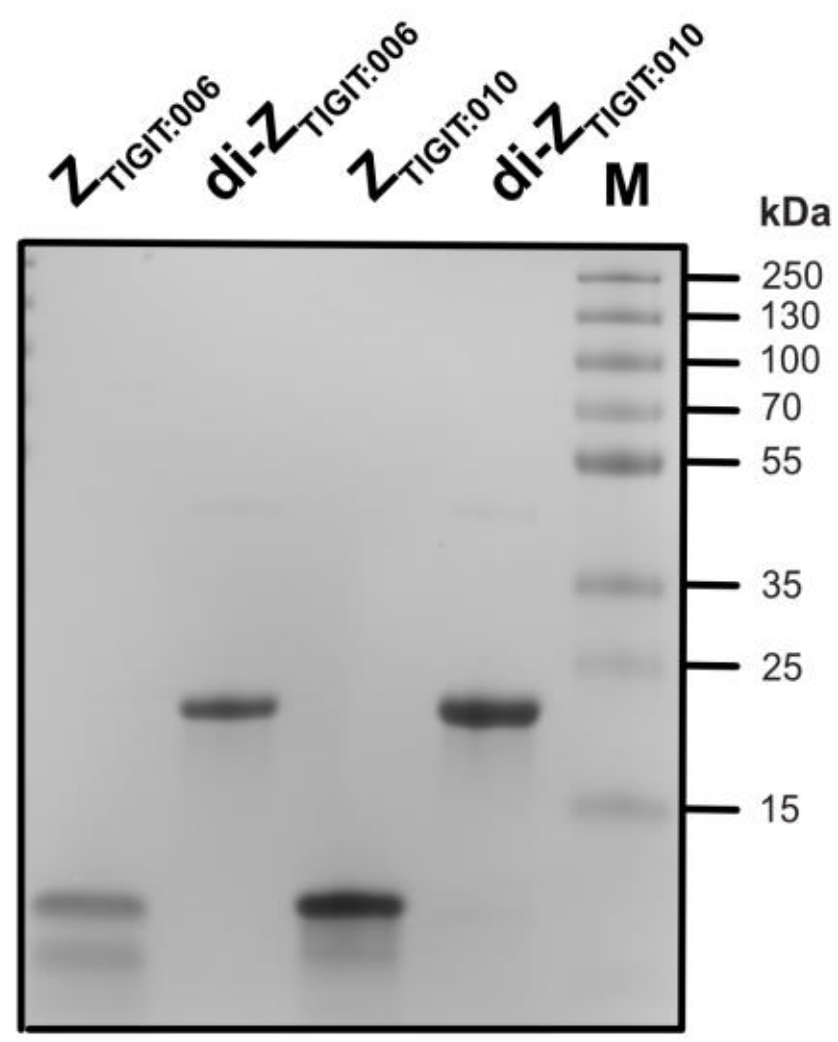

Figure S2. SDS-PAGE analysis of the purified affibody-ABD fusion proteins. The proteins were of essentially the correct molecular weights (14 kDa for the monomers and 21 kDa for the dimers). A protein standard was loaded in lane M with the molecular weights of the standard proteins indicated to the right of the gel.

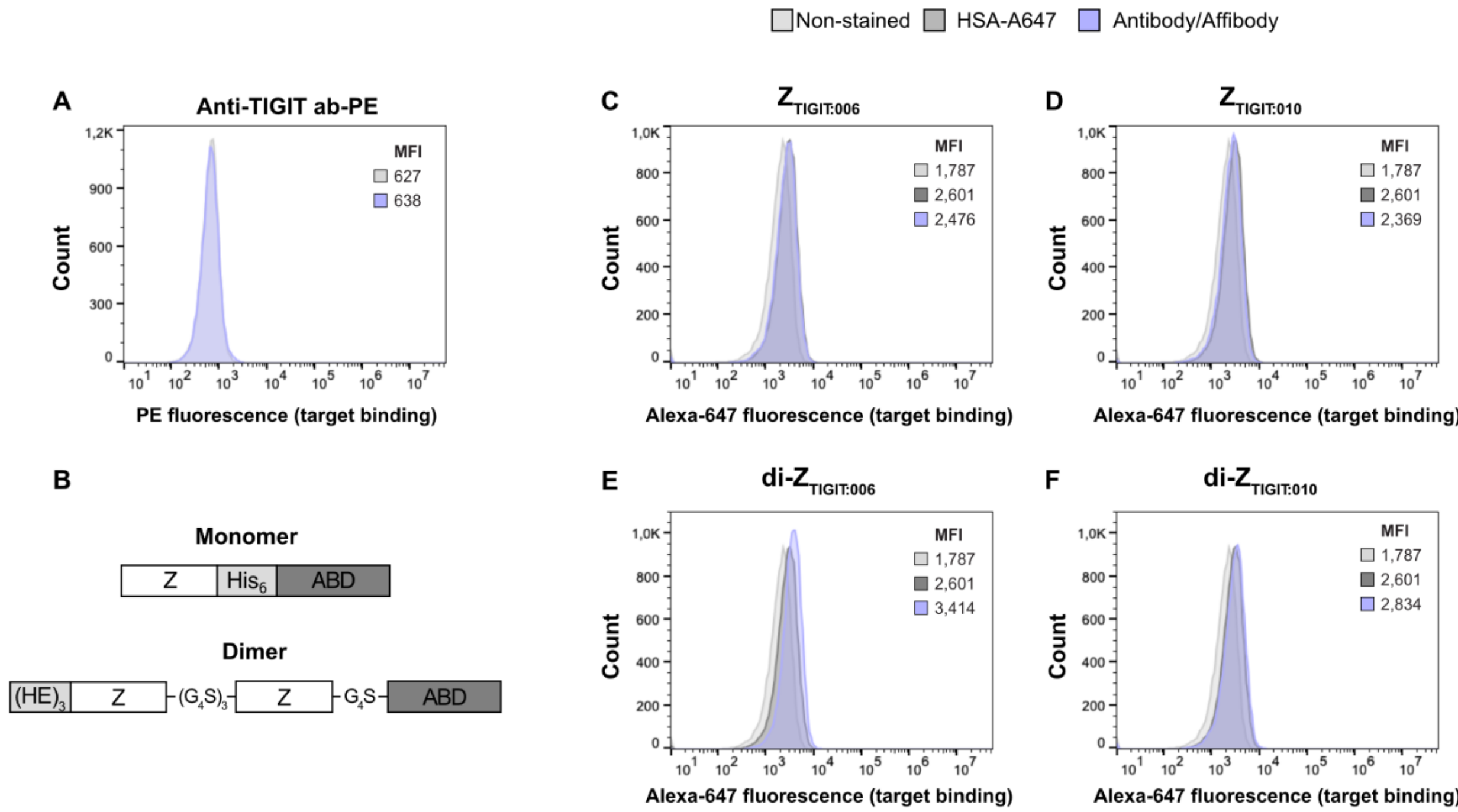

Figure S3. Analysis of specificity by flow cytometry. **A.** Nontransfected HEK293T cells were stained with an anti-TIGIT antibody conjugated to PE (violet) and nonstained cells (grey). **B.** Schematic representation of the monomeric and dimeric constructs with Z indicating affibody molecules. The cysteines in the dimeric constructs were alkylated for the experiment. **C.** and **D.** Nontransfected HEK293T cells stained with $Z_{TIGIT:006}$ or $Z_{TIGIT:010}$ (100 nM) followed by HSA labeled with Alexa-647 (HSA-A647) (violet) as secondary reagent. As controls, nonstained cells (light grey) and cells stained with only the secondary reagent (dark grey) were analyzed. **E.** and **F.** Nontransfected HEK293T cells stained with di-$Z_{TIGIT:006}$ or di-$Z_{TIGIT:010}$ (100 nM, violet) together with HSA-A647 as secondary reagent or with only the secondary reagent (dark grey) and nonstained cells (light grey). Each analysis was performed in duplicates, which essentially overlapped. For clarity, only one of the recorded histograms is presented in the figure. Mean fluorescence intensities (MFI) were calculated for each histogram and are displayed in the panels.

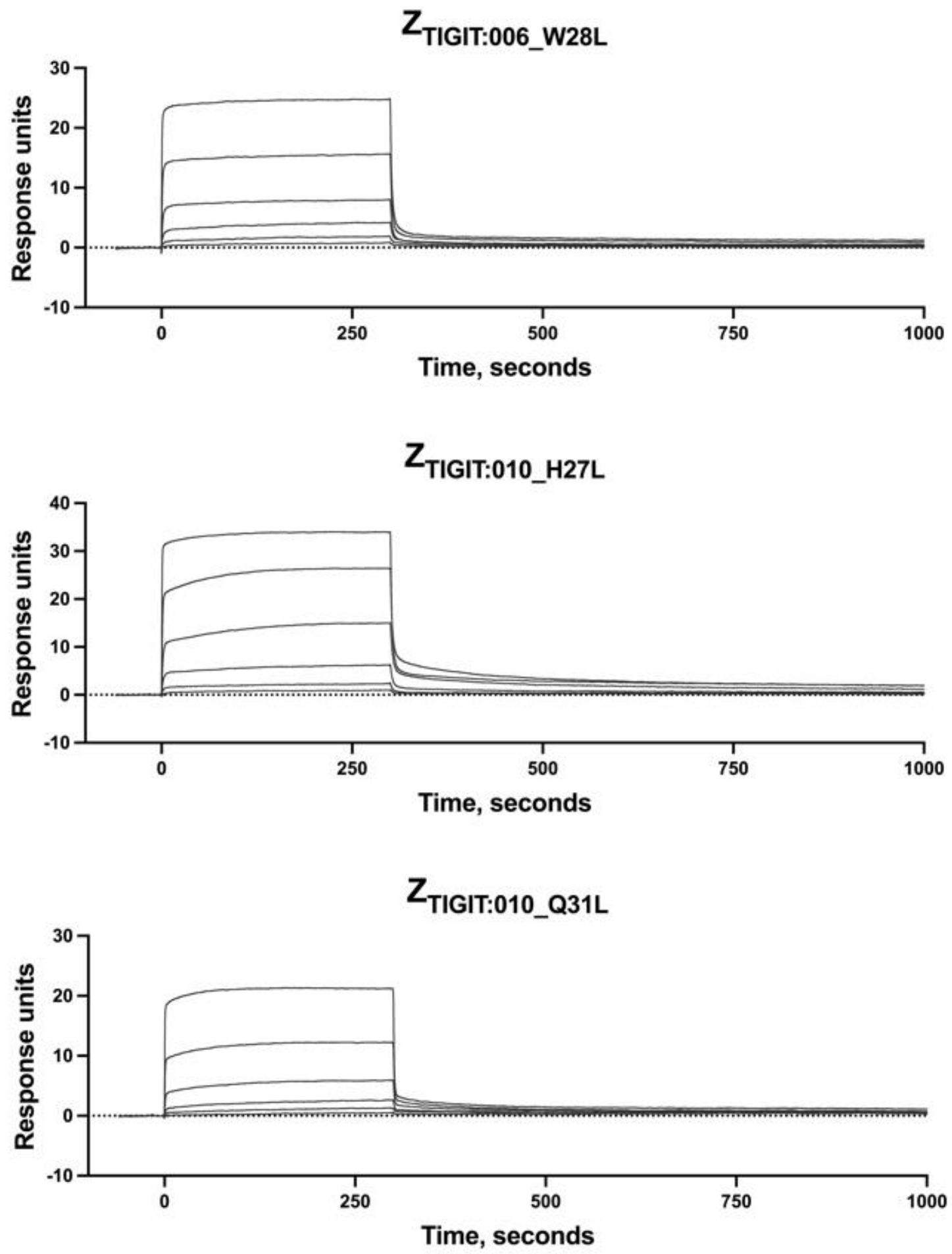

Figure S4. SPR Sensorgrams of three single mutant variants, $Z_{TIGIT:006\_W28L}$, $Z_{TIGIT:010\_H27L}$, and $Z_{TIGIT:010\_Q31L}$. Each panel shows an overlay of the sensorgrams obtained after injection of a dilution series of each affibody candidate over a surface with immobilized TIGIT (ECD). Each concentration was injected in duplicate.

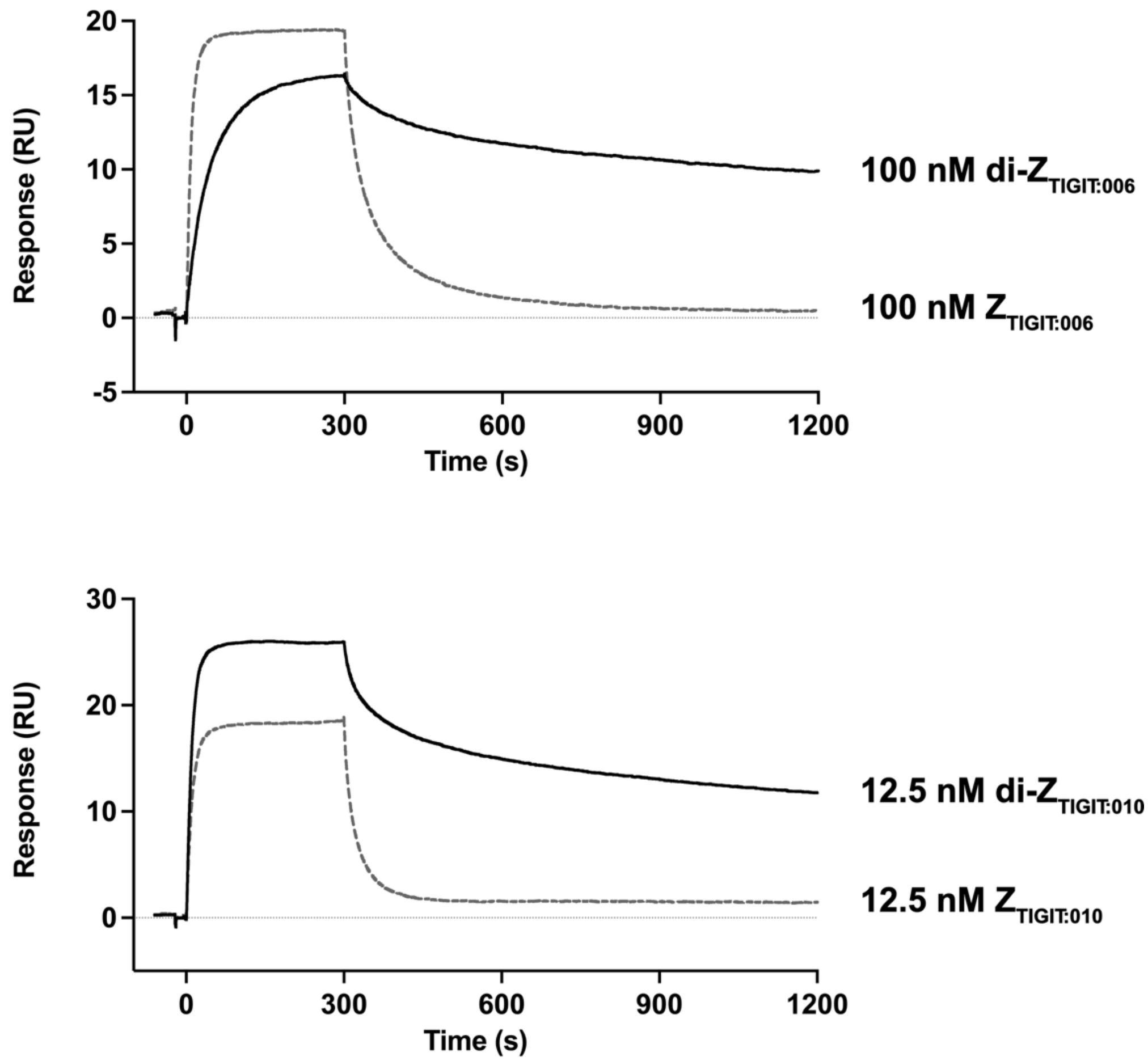

Figure S5. Overlapping SPR Sensorgrams of the monomeric versions and the dimeric versions of $Z_{TIGIT:006}$ and $Z_{TIGIT:010}$ showing slower off-rates, of the dimeric versions.

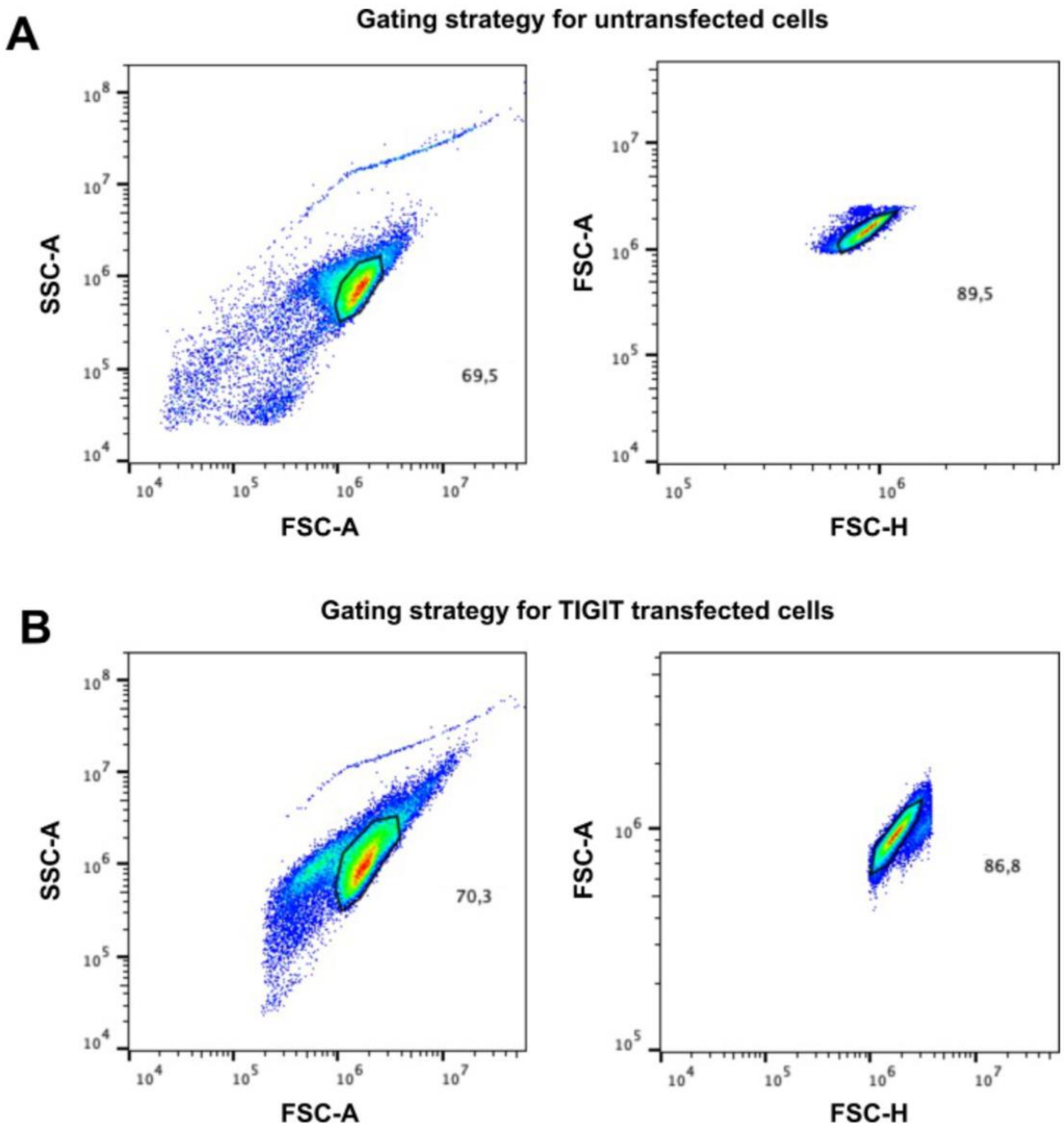

Figure S6. The gating strategy for the flow cytometry analysis presented in Figures 4 and S3. The FSC-A/SSC-A dotplot was first used to separate cells from debris followed by gating for single cells from the FSC-H/FSC-A dotplot. **A.** shows an example of gating nontransfected HEK293T cells and **B.** shows an example of gating transfected cells. All analyses performed on nontransfected HEK293T cells were essentially identical and used the same gates. All analyses performed on TIGIT-expressing HEK293T cells were essentially identical and used the same gates.
